# Laser Frequency Noise in Coherent Optical Systems: Spectral Regimes and Impairments

**DOI:** 10.1038/s41598-017-00868-4

**Published:** 2017-04-12

**Authors:** Aditya Kakkar, Jaime Rodrigo Navarro, Richard Schatz, Xiaodan Pang, Oskars Ozolins, Aleksejs Udalcovs, Hadrien Louchet, Sergei Popov, Gunnar Jacobsen

**Affiliations:** 1 Optics and Photonics Division, KTH Royal Institute of Technology, Electrum 229, SE-16440 Kista, Sweden; 2 Networking and Transmission Laboratory (NETLAB), Acreo Swedish ICT, AB SE-16425 Kista, Sweden; 3 VPIphotonics GmbH, DE-10587 Berlin, Germany

## Abstract

Coherent communication networks are based on the ability to use multiple dimensions of the lightwave together with electrical domain compensation of transmission impairments. Electrical-domain dispersion compensation (EDC) provides many advantages such as network flexibility and enhanced fiber nonlinearity tolerance, but makes the system more susceptible to laser frequency noise (FN), e.g. to the local oscillator FN in systems with post-reception EDC. Although this problem has been extensively studied, statistically, for links assuming lasers with white-FN, many questions remain unanswered. Particularly, the influence of a realistic non-white FN-spectrum due to e.g., the presence of 1/f-flicker and carrier induced noise remains elusive and a statistical analysis becomes insufficient. Here we provide an experimentally validated theory for coherent optical links with lasers having general non-white FN-spectrum and EDC. The fundamental reason of the increased susceptibility is shown to be FN-induced symbol displacement that causes timing jitter and/or inter/intra symbol interference. We establish that different regimes of the laser FN-spectrum cause a different set of impairments. The influence of the impairments due to some regimes can be reduced by optimizing the corresponding mitigation algorithms, while other regimes cause irretrievable impairments. Theoretical boundaries of these regimes and corresponding criteria applicable to system/laser design are provided.

## Introduction

In the 80’s and 90’s, coherent detection was extensively studied because of its ability to provide higher receiver sensitivity compared to non-coherent detection in optical communication systems. However, the commercial success of the erbium doped fiber amplifier (EDFA) around the same period made coherent communication less attractive^1–3^. The need for higher capacity along with the advancement in digital signal processing (DSP) has recently revived both commercial and research interest in coherent optical communications. DSP enabled coherent communication has the ability to increase spectral efficiency, compensate for transmission impairments and obtain carrier tracking in the receiver^4, 5^. However, it is observed that the received signal in coherent optical links, even after DSP processing for dispersion and laser phase noise compensation, remains impaired by an enhancement of noise originating from the frequency noise (FN) of the local oscillator (LO) laser. This enhanced noise is commonly known as equalization enhanced phase noise (EEPN)^6–9^. EEPN is observed in both amplitude and phase domains and it has been concluded that it is difficult to mitigate using simple DSP techniques^7^. Various studies have been performed to evaluate the EEPN impairment in different systems such as single carrier quadrature amplitude modulation (QAM) systems, super-channels and others. All these studies considered only lasers with ideal Lorentzian line shape, i.e. with white FN spectrum^10–16^. We recently revisited the phenomenon for the white FN case. We showed that EEPN induced penalty is mainly due to the low frequency fluctuations of the laser^17^. We also provided and experimentally validated closed form expressions for parameters essential for system design in the presence of EEPN^18, 19^. However, many questions still remain unanswered, particularly the consideration that lasers in general have non-white FN spectrum. The laser FN spectrum is generally non-white due to the presence of 1/f noise and, in the case of semiconductor lasers, also carrier induced noise^20, 21^. Therefore, a more in-depth analysis considering a laser with a general non-white FN spectrum compared to the previously provided statistical analysis, is needed.

In this paper, we provide the non-linear time variant (non-LTI) analysis of coherent optical links utilizing a general laser with non-white FN spectrum. The analysis reveals that depending on the dispersion induced pulse broadening seen by the laser, for a given system specification, its FN spectrum can be divided into multiple regimes. In each regime different FN induced impairments occur. The influence of these impairments can either be reduced by optimizing the corresponding DSP algorithms or by fulfilling the design criteria given in this paper. The findings of the analysis are validated with numerical simulations and system experiments.

## Results

### Theory

Consider a laser with temporal phase evolution $\varphi (t)$ and instantaneous frequency $f(t)=(1/2\pi )\cdot d\varphi (t)/dt$. Then, the phase evolution and instantaneous frequency evolution functions over a certain observation period *τ* around the time instant *t*_*0*_ can be written as

1 $${\varphi }_{\tau }(t^{\prime} )=\varphi (t^{\prime} )\,\,and\,{f}_{\tau }(t^{\prime} )=f(t^{\prime} )\,\,\,\,\,\,t^{\prime} \in [{t}_{0}-\frac{\tau }{2},{t}_{0}+\frac{\tau }{2}]$$

The phase evolution over the period *τ* can be rewritten in terms of mean frequency ${f}_{\tau ,mean}({t}_{0})={E}_{t}[{f}_{\tau }(t^{\prime} )]$, where E_τ_[] is the mean operator over period τ as

2 $${\varphi }_{\tau }(t^{\prime} )=2\pi {f}_{\tau ,mean}({t}_{0})t^{\prime} +{\varphi }_{\tau }^{res}(t^{\prime} )\,\,\,\,\,\,\,t^{\prime} \in [{t}_{0}-\frac{\tau }{2},{t}_{0}+\frac{\tau }{2}]$$

where ${\varphi }_{\tau }^{res}(t^{\prime} )$ corresponds to the residual phase around the phase given by the mean frequency. Therefore, the laser output ${x}_{\tau }(t^{\prime} )$ during the period of observation *τ* around the time instant *t*_*o*_ is given by,

3 $${x}_{\tau }(t^{\prime} )=A{e}^{j{\varphi }_{\tau }(t^{\prime} )}=A{e}^{j\{2\pi {f}_{\tau ,mean}({t}_{0})t^{\prime} +{\varphi }_{\tau }^{res}(t^{\prime} )\}}\,\,\,\,t^{\prime} \in [{t}_{0}-\frac{\tau }{2},{t}_{0}+\frac{\tau }{2}]$$

${\varphi }_{\tau }^{res}(t^{\prime} )$ in equations (2–3) contains all fluctuations faster than 1/*τ* and has zero mean over the observation period. While fluctuations slower than 1/τ are contained in ${f}_{\tau ,mean}({t}_{0})$ during this period of observation. Thus, the amplitude spectrum over the observation period *τ* can be written as,

4 $${X}_{\tau }(f,{t}_{0})=A\delta (f-{f}_{\tau ,mean}({t}_{0}))\otimes {X}_{\tau }^{res}(f,{t}_{0})=A{X}_{\tau }^{res}(f-{f}_{\tau ,mean}({t}_{0}),{t}_{0})$$

In equation (4) ${X}_{\tau }^{res}(f,{t}_{0})$ is the Fourier transform of the laser output due to frequency fluctuations faster than 1/τ.

**Figure 1:**
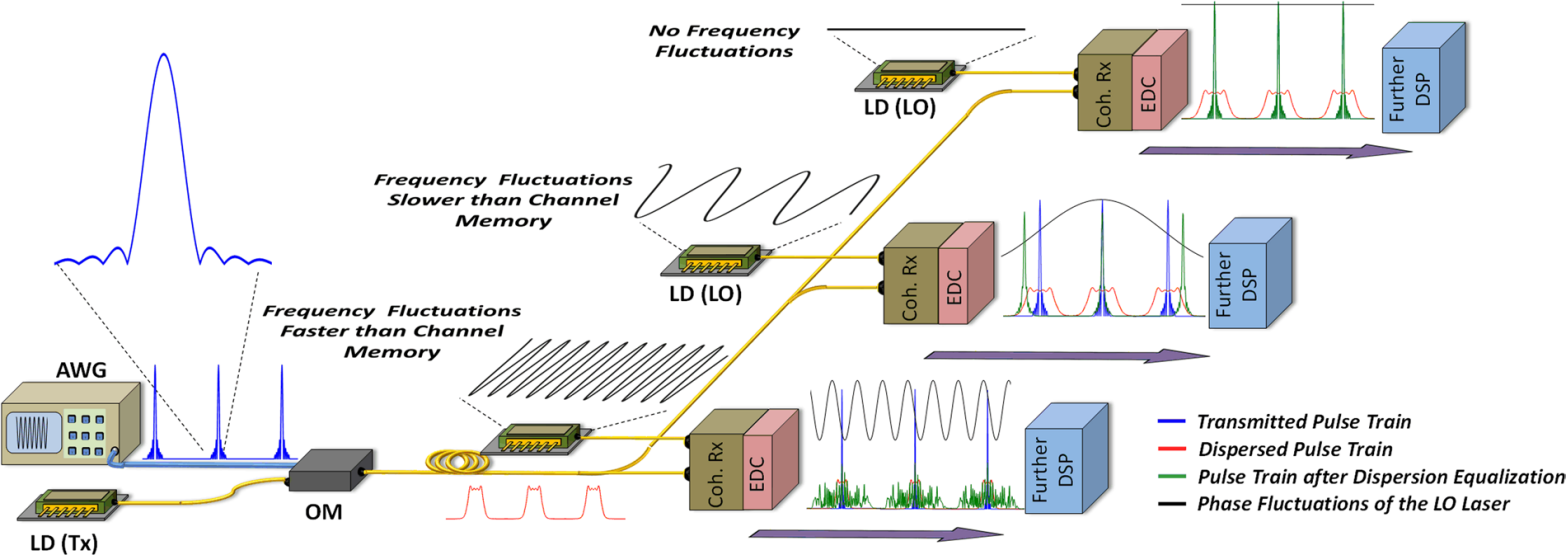
Qualitative representation of the phenomenon. The envelop of On-Off keying encoded pulse train of Nyquist pulses after the electrical transmitter is shown in blue. The dispersed pulse train after optical modulation and transmission over fiber is shown in red. The phase of the LO laser is shown in black. The received pulse train envelope after EDC is shown in green superimposed with the transmitted and dispersed pulse train neglecting the latency of the fiber. AWG: Arbitrary Waveform Generator, LD (Tx): Laser Diode (Transmitter), OM: Optical Modulator, LD (LO): Laser Diode (Local Oscillator), Coh. Rx.: Coherent Receiver, EDC: Electronic Dispersion Compensation, DSP: Digital Signal Processing.

The above discussion becomes important in the context of coherent optical communication links with electrical domain dispersion compensation (EDC). There exist multiple alternatives to perform all electronic impairment mitigation^22^. In this section, without loss of generality, we will consider the most commonly used system configuration with post reception impairment mitigation using digital signal processing. The other configurations are discussed in the Discussion and Conclusion section utilizing the results obtained in this section. We utilize the base band equivalent representations of the components to perform non-linear time variant analysis of the coherent optical system with post reception EDC followed by processing for impairments such as laser phase noise (see Fig. 1 below).

In the following analysis, again without loss of generality, we consider a power normalized representation of the components. This is done in order to ensure that the net system gain remains unity irrespective of the transmitted constellation. In general, the incoming bit stream is translated into a band-limited complex electrical signal, represented by the Fourier transform pair ${r}_{elec}(t)|{R}_{elec}(f)$, in the electrical transmitter. This band limited signal is then modulated on a laser carrier to generate the transmitted signal. The stochastic base band equivalent representation of the transmitting (Tx) laser can be written with the Fourier Transform pair ${e}^{j{\varphi }_{Tx}(t)}{|X}_{Tx}(f)$. The optical transmitter output is then transmitted over the all-pass dispersive fiber with response ${h}_{f}(t)|{e}^{jk{f}^{2}}$. The accumulated dispersion factor is defined as $k=2\cdot {\pi }^{2}\cdot {\beta }_{2}\cdot l$, where, *β*_*2*_ is the group velocity dispersion parameter (GVD) and *l* is the fiber length. At the receiver end, the optical signal is coherently received with an LO laser having stochastic base band equivalent spectrum ${e}^{j{\varphi }_{LO}(t)}{|X}_{LO}(f)$. The detected signal is sampled at the Nyquist rate or higher in a practical system and followed by chromatic dispersion equalization. EDC can be modeled as the inverse channel response function ${h}_{f}^{-1}(t)|{e}^{-jk{f}^{2}}$. The dispersion equalized signal is given by $r^{\prime} (t)|R^{\prime} (f)$. The signal is then processed for mitigation of other impairments such as carrier phase noise and the transmitted bits are recovered. It is important to note that convolution and multiplication possess non-associative and non-commutative relationship. Thus, the operations need to be performed in the order of their physical occurrence. We start our analysis in frequency domain. The received signal *R*′(*f*) after the electronic dispersion compensation is given by

5 $$R^{\prime} (f)=[R(f)\cdot {e}^{jk{f}^{2}}\otimes {X}_{LO}(f)]\cdot {e}^{-jk{f}^{2}}$$

Performing the operations, in the order of physical occurrence we get,

6 $$R^{\prime} (f)={\int }_{-\infty }^{\infty }R(f-f^{\prime} )\cdot {e}^{jk({f^{\prime} }^{2}-2ff^{\prime} )}\cdot {X}_{LO}(f^{\prime} )df^{\prime} $$

Utilizing equation (6) and the steps detailed previously^18^, the time domain response for the bandlimited electrical signal ${r}_{elec}(t)=\sum _{n}{r}_{pulse}^{n}(t)$ composed of pulses ${r}_{pulse}^{n}(t)$, after the EDC is then given by

7 $$\begin{array}{rcl}r^{\prime} (t) & = & \sum _{n}{\int }_{-\infty }^{\infty }{r}_{pulse}^{n}(t-\frac{kf^{\prime} }{\pi }-\frac{k{f}_{\tau ,mean}[n]}{\pi }){X}_{\tau ,LO}^{res,n}(f^{\prime} )\\  &  & \cdot {e}^{j{\varphi }_{Tx}(t-\frac{kf^{\prime} }{\pi }-\frac{k{f}_{\tau ,mean}[n]}{\pi })}\cdot {e}^{jk\{{f^{\prime} }^{2}+{f}_{\tau ,mean}^{2}[n]+2f^{\prime} {f}_{\tau ,mean}[n]\}}\cdot {e}^{j2\pi (f^{\prime} t+{f}_{\tau ,mean}[n]t)}df^{\prime} \end{array}$$

In equation (7), ${X}_{\tau ,LO}^{res}(f)$ is the LO laser frequency spectrum due to the residual fluctuations around the mean ${f}_{\tau ,mean}[n]$ over the observation period. The observation period is the channel memory (CM) that determines the broadening of the signal after the dispersive fiber. Further, it is interesting to note that the Tx laser observes no dispersion induced pulse broadening. Hence, the FN of the Tx laser only causes phase noise impairment in this system configuration. Further, in the special case of ideal optical dispersion compensation, the accumulated dispersion factor *k* = 0. Then the symbol observes the LO phase noise over the observation period equal to the symbol period *T*_*s*_ and the LO phase noise also appears only as a phase noise impairment.

In the specific case of coherent optical QAM systems, the incoming bits are mapped onto symmetrically distributed symbols *c*_*n*_ in the complex plane. This results in a symbol train at the baudrate *R*_*b*_ */m*, where *m* is the number of bits encoded per symbol and *R*_*b*_ is the incoming bit rate. Pulse shaping is performed on this symbol train with symbol period *T*_*s*_ *= m/R*_*b*_ to generate a band-limited continuous signal. The pulse shaping filter can be represented by the Fourier transform pair ${h}_{ps}(t)|{H}_{ps}(f)$. Plugging ${r}_{elec}(t)=\sum _{n}{c}_{n}{h}_{ps}(t-n{T}_{s})$ in equation (7) we get, after the EDC,

8 $$\begin{array}{rcl}r^{\prime} (t) & = & \sum _{n}{\int }_{-\infty }^{\infty }{c}_{n}\cdot {h}_{ps}(t-n{T}_{s}-\frac{kf^{\prime} }{\pi }-\frac{k{f}_{\tau ,mean}[n]}{\pi }){X}_{\tau ,LO}^{res,n}(f^{\prime} )\\  &  & \cdot {e}^{j{\varphi }_{Tx}(t-\frac{kf^{\prime} }{\pi }-\frac{k{f}_{\tau ,mean}[n]}{\pi })}\cdot {e}^{jk\{{f^{\prime} }^{2}+{f}_{\tau ,mean}^{2}[n]+2f^{\prime} {f}_{\tau ,mean}[n]\}}\cdot {e}^{j2\pi (f^{\prime} t+{f}_{\tau ,mean}[n]t)}df^{\prime} \end{array}$$

The channel memory depends on the system specifications such as accumulated dispersion, signal baudrate amongst others. In the case of a Nyquist pulse shaped signal with a given *Rolloff* the channel memory can approximately be written as^23^ ${\tau }_{CM}\approx (k\cdot Baudrate\cdot \{1+Rolloff\})/\pi$.

The implications of equations (7 and 8) and the preceding discussion are qualitatively depicted in Fig. 1. For the purpose of illustration, we consider the envelop evolution of an on-off encoded train of Nyquist pulses with *Rolloff* = 0.15. The train of Nyquist pulses (shown in blue) are generated by the electrical transmitter and modulated on the Tx laser carrier. After transmission over dispersive fiber the pulses suffer dispersion induced broadening (depicted in red). In order to illustrate the impact of the FN of the LO, we study how this dispersed signal is coherently detected with three different LO lasers having sinusoidal frequency fluctuations (for depiction purpose). For the sake of the illustration, the latency due to fiber transmission, which is same in the three reception scenario, is neglected. In the first case, with a laser with no frequency fluctuations (depicted by zero phase evolution in black). In this case both the mean frequency ${f}_{\tau ,mean}$ over the observation period and the frequency fluctuation resulting in residual phase around the phase due to mean frequency are zero. Thus in eqs (7) and (8) the LO frequency response can be replaced by $\delta (f)$ and there is no EEPN phenomenon. Thus the transmitted pulse train is recovered after EDC (in green), perfectly overlapping the transmitted one. The second case corresponds to a LO laser having frequency fluctuations slower than channel memory. Each recovered pulse after EDC in the pulse train is then, as depicted in Fig. 1, delayed by an unequal amount as predicted by equations (7–8). The observed delay given by $\Delta {\tau }_{mean}=\frac{k{f}_{\tau ,mean}}{\pi }$ in eqs (7) and (8) is proportional to the slope of the phase modulation (and hence the mean frequency) seen by the broadened symbol over the observation time. As could be seen in Fig. 1, the central pulse observes almost no delay while the side pulses observe positive or negative delay depending on whether they see the rising or falling edge of the phase. In the third and last case, the effect of fluctuations faster than ${\tau }_{CM}^{-1}$ is depicted. The frequency fluctuations within the broadened symbol period translate into the demodulation of multiple delayed versions of the pulse with delay given by $\frac{kf^{\prime} }{\pi }$ in eqs (7) and (8). These delayed versions interfere to cause inter and intra symbol interference as given by the integral over f’ in eqs (7) and (8). Thus, statistically all symbols observe the same amount of frequency modulation. In reality when the frequency fluctuations faster and slower than CM co-exist, both timing drift, timing jitter and inter and intra symbol interference will be present.

A FN spectral component at $1/({\tau }_{CM}+n{T}_{s}/2)$ will cause maximum timing fluctuation $\Delta {\tau }_{mean}[n]-\Delta {\tau }_{mean}[0]$ between the present (0^th^) and the n^th^ symbol. Note that since the timing drift is due to movement of the individual symbols and not the sampling clock, this phenomenon also results in inter-symbol interference. Hence, with the increase in the speed of the fluctuations of the carrier frequency the timing drift converts into timing jitter and finally into irretrievable inter and intra symbol interference for fluctuations above the channel memory as elaborated in Fig. 1. The relative time delay $\frac{\Delta {\tau }_{mean}}{{T}_{s}}=\frac{k{f}_{\tau ,mean}}{\pi {T}_{s}}$ i.e. time delay as fraction of the symbol period, can be used as figure of merit in practical systems to estimate the system impairments.

**Figure 2:**
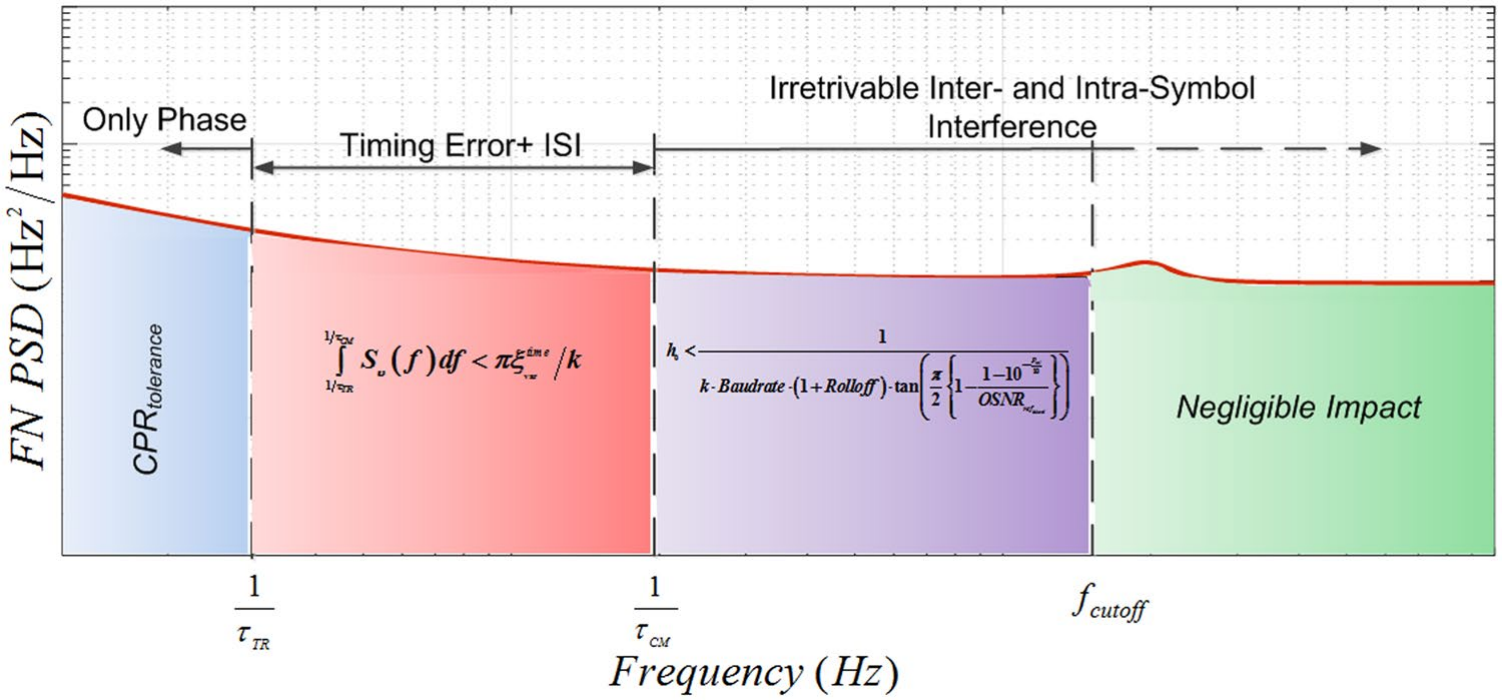
Regime segmentation of frequency noise spectrum. *CPR*_*tolerance*_: Carrier Phase Recovery tolerance, *1/τ*_*TR*_: Timing Recovery Bandwidth, *τ*_*CM*_: Dispersion Channel Memory, *f*_*cutoff*_: Cut off frequency.

To illustrate more in detail how the frequency noise affects the system performance, the FN spectrum can be divided into various regimes as shown in Fig. 2. We assume that all FN processes are Gaussian distributed and thus the probability of deviation beyond a certain limit is determined by the FN variance, given by the integral of the FN power spectral density. The first frequency noise regime is given by $f < 1/{\tau }_{TR}$ where $1/{\tau }_{TR}$ is the timing recovery bandwidth. The timing recovery bandwidth depends on the timing recovery method along with the system operational specifications, such as signal OSNR amongst others.

In this regime timing fluctuations slower than the timing recovery bandwidth are ideally tracked by the timing recovery algorithm and the only dominant impairment is the phase drift. Either the carrier phase recovery (CPR) algorithm needs to be optimized to overcome this impairment or the FN spectrum should meet the following criteria in terms of maximum tolerable FN variance for CPR (CPR_tolerance_)

9 $${\int }_{0}^{1/{\tau }_{TR}}{S}_{\upsilon }(f)df < CP{R}_{Tolerance}$$

For the next FN regime $1/{\tau }_{TR} < f < 1/{\tau }_{CM}$, the bandwidth of the timing recovery algorithm along with the adaptive filter design can be optimized to minimize the impairment apart from the carrier phase recovery. Alternatively, the system can be designed such that the following criteria in terms of tolerable timing error variance ${\varepsilon }_{\text{variance}}$ is satisfied

10 $${\int }_{1/{\tau }_{TR}}^{1/{\tau }_{CM}}{S}_{\upsilon }(f)df < \pi {\varepsilon }_{\text{variance}}/k$$

The FN in the regime $f > 1/{\tau }_{CM}$ results in irretrievable inter and intra symbol interference. For this regime, there exists no DSP optimization to minimize the impact. The performance in these regimes can be improved by either mitigating the FN using hardware based techniques or choosing a laser meeting the criteria below^18, 19^,

11 $${h}_{0} < \frac{1}{k\cdot Baudrate\cdot (1+Rolloff)\cdot \,\tan (\frac{\pi }{2}\{1-\frac{1-{10}^{-\frac{{P}_{tol}}{10}}}{OSN{R}_{re{f}_{inband}}}\})}$$

In equation (11), *h*_*0*_ is the maximum tolerable white frequency noise level above the channel memory to limit the system penalty below *P*_*tol*_ (dB) as derived and validated in refs 18 and 19. *Baudrate* and $OSN{R}_{re{f}_{inband}}$ are the symbol rate and the reference in band optical signal to noise ratio (OSNR). In these regimes, the impact falls off inversely proportional to the weighting factor ${X}_{\tau ,LO}^{res}(f)$ in equations (7–8) which falls off inversely proportional to frequency *f* as shown in ref. 17. Thus, an upper cut-off frequency *f*_*cutoff*_ can be considered beyond which the impact of frequency noise can be neglected similar to the one derived for white frequency noise in ref. 18.

### Numerical Simulations

**Figure 3:**
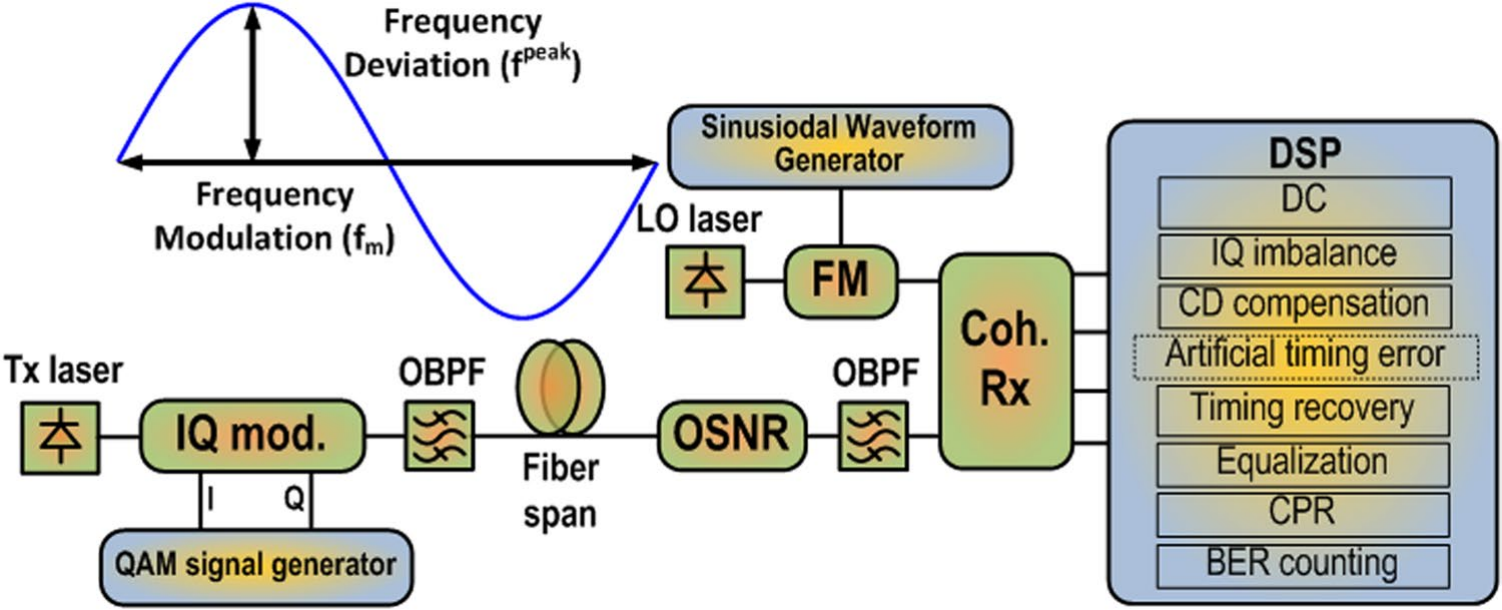
Simulation Setup. Tx Laser: Transmitting Laser, QAM: Quadrature Amplitude Modulation, IQ mod.: In-phase and Quadrature-phase modulator, OBPF: Optical Band Pass Filter, OSNR: Optical Signal to Noise Ratio loading module, FM: Frequency Modulator, LO laser: Local Oscillator Laser, DSP: Digital Signal Processing.

In the previous section, the theory concludes that slow frequency fluctuations of the LO laser causes timing drift and jitter whereas fast frequency fluctuations cause inter and intra symbol interference. Timing jitter tolerance, in digital fiber optic systems, is conventionally defined in terms of sinusoidal jitter whose amplitude, when applied to an equipment input, causes a designated degradation in error performance^24, 25^. Hence, in order to validate our conclusion that slow frequency fluctuations of the LO have similar effect on the system as timing jitter in the clock, we performed simulations where the frequency of the LO laser was modulated sinusoidally, using the setup shown in Fig. 3. The details are provided in the Methods section. The amplitude and frequency of the sinusoidal modulation is swept in order to characterize the system. The timing recovery is performed employing the full received signal resulting in same timing phase for all symbols in the received signal.

**Figure 4:**
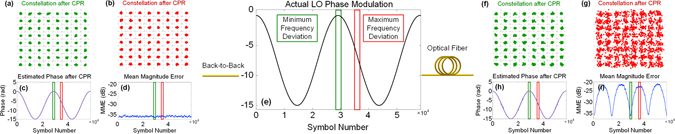
Qualitative analysis of the impact of LO frequency deviation in 28 Gbaud 64-QAM system with and without fiber transmission for peak LO frequency deviation of 7.2 MHz at 1 MHz modulation frequency and 35 dB OSNR. (**a,b**) Received constellation for the symbols seeing minimum and maximum frequency deviation in back to back transmission, respectively. (**c,d**) Estimated phase in violet and MME in blue of the received signal in back to back transmission, respectively. (**e**) Actual phase modulation of the LO. (**f,g**) Received constellation for the symbols seeing minimum and maximum frequency deviation after fiber transmission, respectively. (**h,i**) Estimated phase in violet and MME in blue of the received signal after fiber transmission, respectively.

Figure 4 depicts the qualitative comparison between the reception of a 28 Gbaud 64-QAM signal with a LO laser modulated with sinusoidal frequency fluctuations in two transmission scenario. In Fig. 4 the peak frequency deviation and the modulation frequency of the LO frequency modulation are 7.2 MHz and 1 MHz, respectively. The two scenarios correspond to a back to back (b2b) transmission (left) and (right) a transmission over 2500 km of standard single mode fiber (SSMF). For the sake of a fair comparison and to ensure that the system performance is EEPN limited, the received OSNR is kept the same at 35 dB for both cases. Three different measures are utilized for the qualitative comparison. The first measure is the received constellation after carrier phase recovery (depicted in Fig. 4a,b,f,g in green for minimum frequency deviation and red for maximum frequency deviation). Second measure is the mean magnitude error (MME) (shown in Fig. 4d,i in blue). MME is defined as the mean magnitude distance between the received symbols with respect to the ideal transmitted symbols. The MME is an analogous measure of the error vector magnitude (EVM), but it considers the symbol displacement only in the radial direction in the complex plane. It isolates the different impairments due to EEPN from the normal phase noise. The third measure is the estimated phase by the carrier phase recovery (shown in Fig. 4c,h in violet). The depicted received constellations in Fig. 4 correspond to the symbols contained within the highlighted windows illustrated in the MME and CPR estimated figures in red and green colors. It can be seen from Fig. 4(d) that in the case of back to back transmission no oscillations are observed in the MME. While in the case of fiber transmission, the MME oscillates with a period equal to half the period of the sinusoidal frequency modulation as seen in Fig 4(i). This is because in this case, symmetrical phase modulation results in equal and opposite delay of the symmetric pulse shaped signal which translate into an equal amount of MME. The impact of fiber transmission can be further appreciated by considering two extreme cases of minimum frequency deviation (highlighted in green) and maximum frequency deviation (highlighted in red). According to theory, symbol suffers a time displacement from the ideal timing phase/sampling instant, given by $\Delta {\tau }_{mean}$ proportional to mean frequency ${f}_{\tau ,mean}$ over the broadened symbol period, as could be seen from eqs (7) and (8). In the case of no fiber transmission, the accumulated dispersion factor k = 0. Hence, irrespective of the region of observation, the phase modulation has no impact on the MME as shown in Fig. 4d. This results in an almost ideal recovery of both the phase and the corresponding constellation (see Fig. 4c and Fig. 4a,b respectively). In the case of fiber transmission, the group of broadened symbols which see minimum frequency deviation (highlighted in green) suffer from minimum or no symbol displacement resulting in an MME dip. Thus, in this case, the symbols are dominantly impaired only by phase noise which is compensated by the CPR (see Fig. 4h,e). This results in the recovery of the symbols as in the back to back case (see Fig. 4a,f). The broadened symbols which observe maximum frequency deviation (highlighted in red), suffer from maximum delay and translate into maximum MME. This timing impairment translates into constellation distortions as shown in Fig. 4g. The CPR, as in the back to back case, is able to recover the phase with some minor distortions when the frequency deviation is maximum.

**Figure 5:**
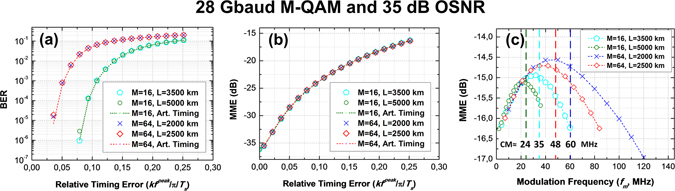
(**a**) BER vs. Relative Timing Error for 28 Gbaud M-QAM transmission (M = 16 and 64) over 3500/5000 km and 2000/2500 km SMF fiber respectively compared to artificially induced timing at 35 dB OSNR and 1 MHz modulation frequency, (**b**) MME vs. Relative Timing Error for the same specification, (**c**) MME vs. Modulation Frequency for peak relative timing error of 0.25 at 35 dB OSNR for same specification. CM shows the theoretical channel memory for different configurations.

Figure 5 presents an analysis considering 28 Gbaud 16-QAM transmission over 5000 km and 3500 km SSMF and 64-QAM transmission over 2500 km and 2000 km SSMF scenarios. For the sake of a fair comparison and to ensure that system performance is EEPN limited, the received OSNR is kept constant during the analysis at 35 dB. In Fig. 5a,b, the bit error ratio and the MME are respectively used as figure of merit for the validation of the relative time delay $\Delta {\tau }_{mean}/{T}_{s}$ derived in the Theory section. The analysis is performed for different LO frequency modulation amplitudes at 1 MHz of modulation frequency as seen in Fig. 5a,b. Results for LO frequency modulation are compared with a system having an equivalent artificial induced sinusoidal timing error. The sinusoidal modulation has, in both the cases, the same frequency and an amplitude that correspond to the same relative peak delay i.e.$\Delta {\tau }_{mean}^{peak}=k{f}_{\tau ,mean}^{peak}/\pi {T}_{s}$ and modulation frequency. It can be observed that for both constellations, the impact of the LO frequency modulation matches closely with the artificially induced timing error. The tolerable timing error decreases with higher order constellations. Figure 5c depicts the variation of the MME as a function of the modulation frequency for a peak relative delay 0.25. The position of the MME peak along the x-axis decreases with the increase in fiber length irrespective of the constellation. This is due to the MME dependence on the channel memory as discussed in the Theory section. For each configuration, the MME increases before the CM and starts falling off after it. The reason for the increase in the MME before the CM and fall thereafter is as follows. All frequency fluctuations slower than the CM contribute to a drift in the mean frequency ${f}_{\tau ,mean}$ between symbols, resulting in a relative delay between them. The separation between the symbols which sees this relative drift is governed by the rate of fluctuations, as shown in the Theory section. The number of symbols experiencing increase in MME increases with the frequency of fluctuations. For a rate of fluctuations $f > 1/{\tau }_{CM}$, a plurality of fluctuation periods occurs within the broadened symbol period and all the symbols statistically see the same amount of fluctuations. The Fourier spectrum of the frequency modulated LO, ${X}_{\tau ,LO}^{res}(f)$, can for sinusoidal modulation be expressed as multiple sidebands with magnitude of *n*-th sideband given by the *n*-th order Bessel function *J*_*n*_(*f* ^*peak*^ */f*). At high frequencies the modulation index, *f* ^*peak*^ */f*, decreases causing the modulation sidebands of the optical LO spectrum to decrease. The received signal output as per eqs (7) and (8) in case of $f > 1/{\tau }_{CM}$ is given by the integral over all delayed versions, each corresponding to a LO sideband, of the demodulated signal. The residual Fourier spectrum of the LO,${X}_{\tau ,LO}^{res}(f)$, can hence be thought of as a weighting factor in eqs (7) and (8) which falls off with the increase in the modulation frequency for the same frequency deviation^17^. Thus the MME also falls off with the increase in modulation frequency.

### Experiments

**Figure 6:**
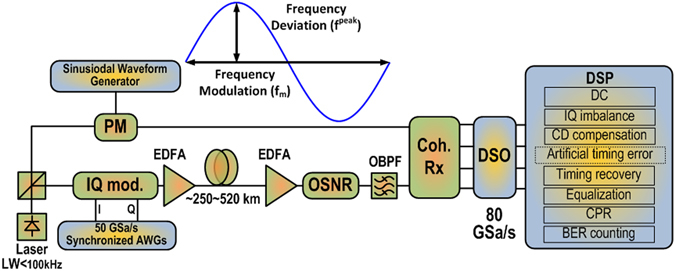
Experimental Setup. AWG: Arbitrary Waveform Generator, EDFA: Erbium Doped Fiber Amplifier, IQ mod.: In-phase and Quadrature-phase modulator, OBPF: Optical Band Pass Filter, OSNR: Optical Signal to Noise Ratio loading module, PM: Phase Modulator, DSO: Digital Sampling Scope, DSP: Digital Signal Processing, *f*_*m*_: Modulation frequency, *f* ^*peak*^: Modulation Amplitude.

**Figure 7:**
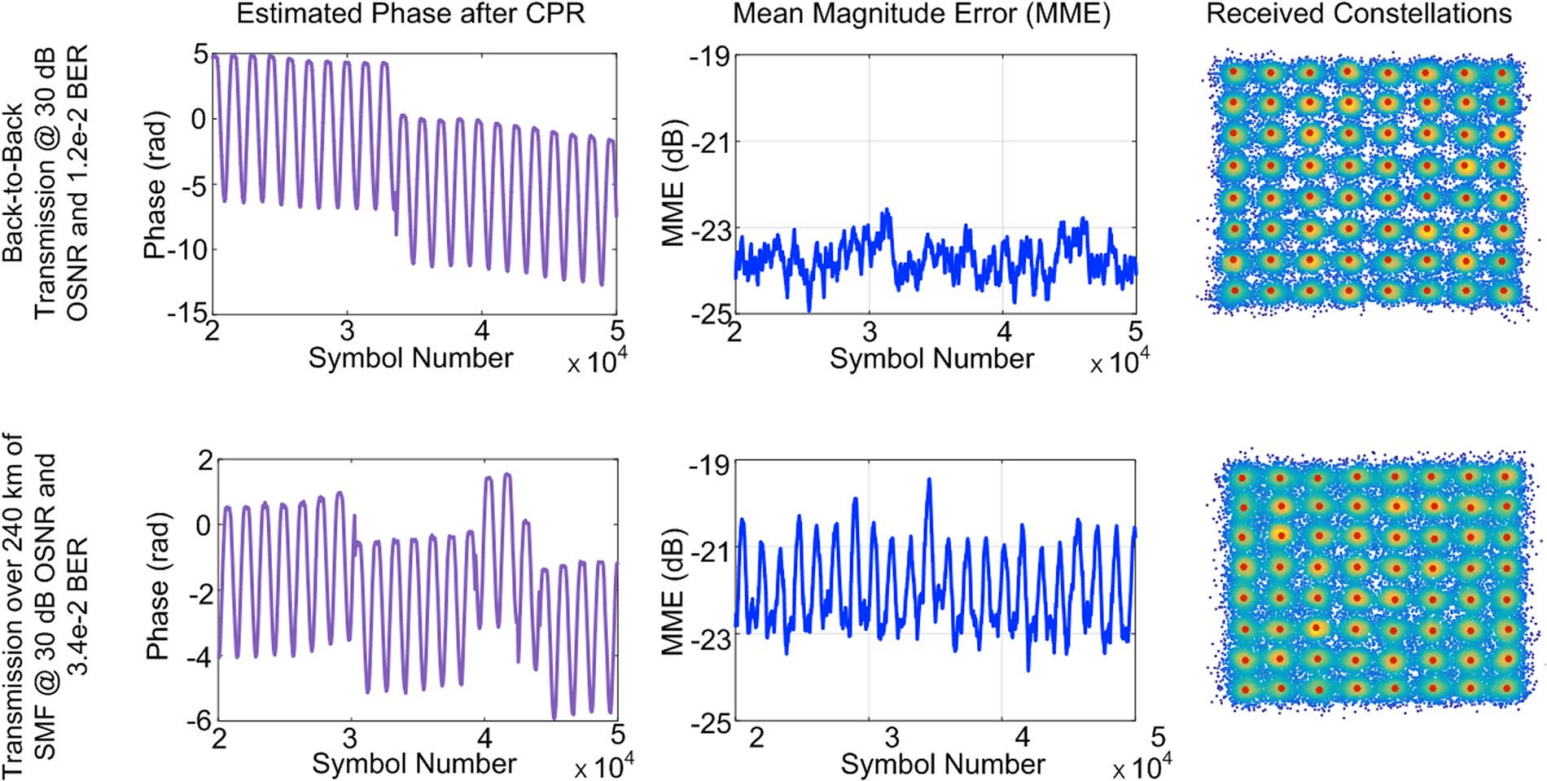
(**a**) Estimated phase noise in violet, mean magnitude error in blue and the received constellation (from left to right) for 28 Gbaud 64-QAM signal for back to back and after transmission over ~250 km of SMF fiber.

The set-up for the experimental validation is depicted in Fig. 6 and details are provided in the Methods section. Figure 7 depicts the qualitative comparison between b2b and after fiber transmission over ~250 km for a 28 Gbaud 64-QAM signal. As expected, it can be seen from Fig. 7 that in the b2b case there is no impact of phase modulation on the MME. The received constellations in this case have only residual phase fluctuations after DSP processing. However, after fiber transmission, periodic oscillations are observed in the MME due to timing error induced by the LO frequency fluctuations. The oscillations have a frequency of 20 MHz (and not expected 40 MHz) which could be attributed to an asymmetry in the modulated phase seen by the signal and averaging in the DSP. Cycle slips after phase recovery were taken care by the use of differential decoding.

**Figure 8:**
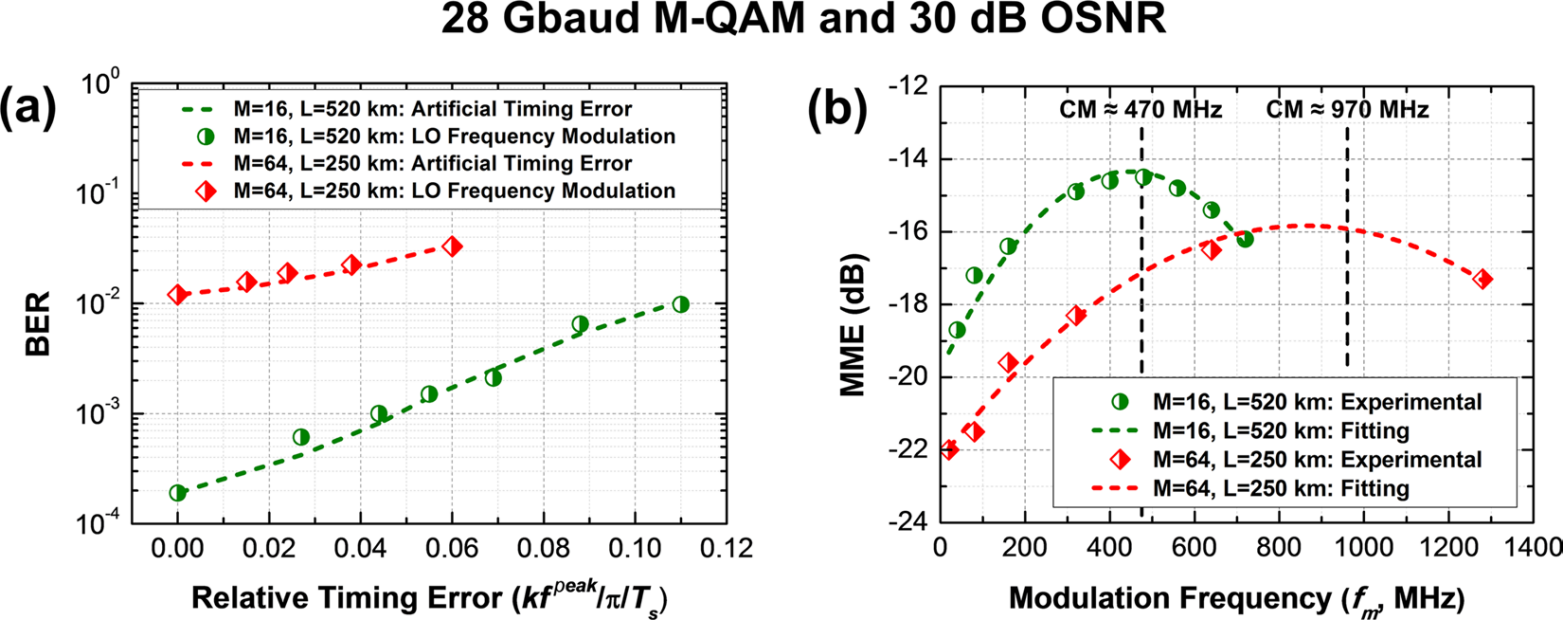
(**a**) BER vs. Relative Timing Error for 28 Gbaud M-QAM transmission (M = 16 and 64) over 520 km and 250 km SMF fiber respectively compared to artificially induced timing at 30 dB OSNR and 20 MHz modulation frequency, (**b**) MME vs. Modulation Frequency for peak relative timing error of 0.11 and 0.06 respectively at 30 dB OSNR for same specification. CM shows the theoretical channel memory for different configurations.

The experimental results for 28 Gbaud 16 and 64-QAM signals after transmission over ~520 km and ~250 km links, respectively, are shown in Fig. 8. The BER versus Peak Relative Delay at 20 MHz modulation frequency for LO frequency modulation amplitude as well as equivalent artificially induced sinusoidal timing error is depicted in Fig. 8(a). As expected for both 16 and 64-QAM links, the BER due to LO sinusoidal phase modulation matches closely with the artificial sinusoidal timing error with same frequency and equivalent relative timing error peak $\Delta {\tau }_{mean}^{peak}/{T}_{s}$ induced in DSP. Figure 8(b) depicts the MME versus LO phase modulation frequency for 16 and 64-QAM with given relative peak delay of 0.11 and 0.06 respectively at 30 dB of OSNR. The timing recovery was performed on the full received signal which resulted in a timing recovery bandwidth ~200 kHz.

In line with the theoretical and numerical analysis in the case of 28 Gbaud 16-QAM signal transmission over ~520 km link, the MME increases with the increase in modulation frequency until the inverse CM (${\tau }_{CM}^{-1}$). This is the regime corresponding to $1/{\tau }_{TR} < f < 1/{\tau }_{CM}$ in Fig. 2 where $1/{\tau }_{TR}$ is ~200 KHz and $1/{\tau }_{CM}$~470 MHz. In this regime the frequency variance is bounded by the maximum tolerable timing error given by eq. (10). The MME, again as expected, starts falling for modulation frequencies from CM onwards, which corresponds to the regime given by $f > 1/{\tau }_{CM}$ in Fig. 2. In this regime the maximum tolerable level of frequency noise variance is given by eq. (11). Similarly, in the case of 28 Gbaud 64-QAM signal transmission over ~250 km link, the MME increases until the frequency ${\tau }_{CM}^{-1}$ of ~970 MHz corresponding to ~250 km transmission and falls off thereafter as expected. Further, depending on the system parameters such as accumulated dispersion and baudrate, a particular frequency fluctuation could be in the $1/{\tau }_{TR} < f < 1/{\tau }_{CM}$ or $f > 1/{\tau }_{CM}$ regimes and, therefore, result in a different set of impairments.

## Discussion and Conclusions

In the results section, we performed the analysis for the most common configuration of coherent fiber optic links. This configuration employs post reception dispersion equalization followed by other signal processing such as timing recovery, carrier phase recovery etc. In this configuration, the design criterion for the Tx laser is the phase noise level tolerated by the phase recovery algorithm in the receiver. The LO laser on the other hand causes, apart from the phase noise, also timing impairment and different design criteria apply for different FN regimes. As discussed in our previous work, there exist more than one configuration for performing all-digital signal processing^20^. The order of the digital signal processing decides whether the Tx laser, LO laser or both causes the presented phenomenon. The analysis in the results section shows that for all schemes, the FN regimes depend on the laser phase noise seen by the dispersion broadened pulse before this broadening compensation. In those regimes, for each laser, the design criteria presented in the result section apply. The results obtained are generic and applicable to other systems such as systems utilizing super channel, non-linear Fourier transform, stokes spaced based direct detection etc.^10–16^.

In conclusion, we have systematically analyzed the impact of lasers having a general frequency noise spectrum in coherent optical links with dispersion compensation in electrical domain. We have shown that the previous statistical understanding is insufficient to fully understand and explain the different impairments caused by the laser FN. Our analysis and results reveals that EEPN impairments are due to inter and intra symbol timing drift and jitter after electrical domain dispersion compensation, induced by the LO frequency noise. Our theoretical and experimental results show that, even in the case of ideal white FN, different regimes of the FN spectrum cause different type of impairments. The impact of the impairments due to FN slower than channel memory can be reduced by optimizing the corresponding DSP algorithm. Only the impairments due to FN above the channel memory are theoretically irretrievable. The results further show that in metro links with standard single mode fiber, the FN mainly translates into timing impairment (apart from phase noise).

We anticipate the impact of our work as follows. First, it provides the fundamental understanding of the influence of laser FN, in any complex coherent system with dispersive channel and electrical dispersion compensation. Second, it provides the design criteria for either dimensioning the system or optimizing DSP routines to increase the impairment tolerance. Finally, in a broader context, it is also useful for designing system utilizing space division multiplexing^16^, in which laser FN imposes a fundamental performance limitation.

## Methods

### Simulation

The simulation setup for the numerical simulation is given in Fig. 3. The simulations are performed using the simulation tool VPItransmissionMaker^TM^ and MATLAB. The data stream is generated using 2^15^–1 pseudorandom bit sequence. This data stream is mapped onto symbols in the complex plane to generate 2^18^ symbols corresponding to 28 Gbaud 16-QAM or 64-QAM signals. This is followed by raised cosine pulse shaping with roll-off factor β = 0.15 to generate a band limited signal. The band limited signal is then modulated on the optical Tx laser, modeled to be ideal, using Mach-Zehnder based in-phase and quadrature phase modulator. The optical signal is then transmitted over single mode fiber (SMF) links, with dispersion coefficient of 16 ps/(nm·km). In order to illustrate the presented phenomenon, fiber non-linearities are neglected. The incoming optical signal before reception is loaded with amplified spontaneous emission noise. This emulates erbium-doped fiber amplifier noise that incrementally adds on to the signal after each fiber span. The receiver consists of a balanced coherent receiver front end and 56 GSa/s analog to digital converters (ADC). The timing jitter tolerance is defined in terms of applied sinusoidal jitter^24, 25^. Thus, the LO laser with sinusoidal frequency fluctuations was emulated to validate the presented theory. The emulation was achieved by frequency modulating an ideal LO laser with a sinusoidal waveform with peak amplitude (frequency deviation) given by *f* ^*peak*^ and modulation frequency given by *f*_*m*_. The data demodulation was performed in MATLAB using the DSP routines described in the Digital Signal Processing Section.

### Experiment

Figure 6 shows the experimental setup. The optical transmitter is composed of two synchronized 50 GSa/s arbitrary waveform generators (AWGs) and an optical IQ modulator. A pseudo-random bit sequence (PRBS15) is Gray mapped onto 28 Gbaud 16 and 64-QAM symbols. This is followed by Nyquist pulse shaping having a 0.15 roll off factor. The sequence is loaded onto the AWGs after resampling in order match the sampling rate of the AWG. The output of the synchronized AWG is fed into an optical IQ modulator. An ECL laser source with less than 100 kHz linewidth is split using a 3 dB polarization maintaining (PolM) splitter. The incoming electrical signal is modulated onto one of the outputs of the PolM splitter using the optical IQ modulator. The output of the IQ modulator, which is either an optical 28 Gbaud 16 or 64-QAM signal, is launched into a ~520 km or ~250 km SSMF transmission link, respectively. An OSNR adjusting module is used for noise loading. It consists of an optical attenuator and an automatic gain control EDFA with constant output power.

The receiver consists of a balanced coherent receiver front end and a digital sampling oscilloscope (DSO). The bandwidth and the sampling rate of the DSO are 33 GHz and 80 GSa/s respectively. The output from the second arm of the PolM splitter is used as the seed for the LO laser. The phase of the LO was modulated sinusoidally to study timing error. The periodic waveform generator is programmed to generate sinusoidal electrical signal with different amplitude and frequency. The output of this device is fed into a phase modulator (PM) to manipulate the frequency/phase of the LO laser sinusoidally in a controlled way, as illustrated in Fig. 8. Data demodulation is performed offline using a DSP routine described in the next section.

### Digital Signal Processing

The digital signal processing routines were performed in Matlab. The received sampled signal from the ADC is fed into the Direct Current (DC) block module to remove the DC component of the received signal. The signal after DC component removal is passed through a low pass filter to remove out of band noise in the received signal. The accumulated dispersion after transmission over standard single mode fiber is compensated using a static frequency domain chromatic dispersion equalizer. Constant modulus algorithm based timing recovery is performed after chromatic dispersion compensation in order to obtain 1 sample per symbol from the oversampled signal. The full received signal block was used for timing recovery. The channel response is then compensated using multi-modulus algorithm based symbol spaced equalizer. Blind phase search (BPS) algorithm was utilized for carrier phase estimation and recovery of the equalized signal. The signal was then differentially decoded followed by bit error ratio (BER) and mean magnitude error calculations. In the case of artificial timing error, the artificial timing error was induced, using a frequency domain time delay induction module before the timing recovery module.
